# Enhanced bioluminescence imaging of tumor cells surviving chemotherapy in a murine model of triple-negative breast cancer

**DOI:** 10.1038/s41523-025-00795-y

**Published:** 2025-07-30

**Authors:** Silvia Steinbauer, Jamie D. Cowles, Mohammad Ali Sabbaghi, Marle Poppelaars, Azaz Hussain, Marina Wagesreither, Daniela Laimer-Gruber, Jozsef Tovari, Gergely Szakacs, Agnes Csiszar

**Affiliations:** 1 Center for Cancer Research, Medical University of Vienna, Vienna, Austria; 2 Department of Biomedical Imaging and Image-Guided Therapy, Division of Structural and Molecular Preclinical Imaging, Medical University of Vienna, Vienna, Austria; 3 Department of Experimental Pharmacology and the National Tumor Biology Laboratory, National Institute of Oncology, Budapest, Hungary

**Keywords:** Breast cancer, Cancer therapeutic resistance, Chemotherapy, Cancer imaging, Cancer models

## Abstract

Triple-negative breast cancer (TNBC) is associated with poor prognosis and high recurrence, driven by residual tumor cells that survive chemotherapy. To monitor therapy response in vivo, we established a clinically relevant TAC regimen (docetaxel, doxorubicin, cyclophosphamide) in mice bearing mammary tumors derived from *K14cre;Brca1*^*F/F*^*;Trp53*^*F/F*^ (KB1P) organoids expressing an mCherry-AkaLuc dual reporter (mCA-KB1P). AkaLuc bioluminescence imaging (AkaBLI) enabled non-invasive detection of minimal residual disease (MRD) with a sensitivity of approximately 1000 cells. As AkaLuc elicited an immune response, we generated Histon2B-mCherry-expressing KB1P organoids (HmC-KB1P) to study tumor cell survival in immunocompetent hosts. Flow cytometry and histological analysis revealed that MRD in immunocompetent mice is characterized by few residual cells with transient loss of epithelial markers, in contrast to immunodeficient hosts, which retains more epithelial-like cells. These findings validate AkaBLI for sensitive MRD detection and highlight the immune system’s critical role in modulating residual tumor cell fate following chemotherapy.

## Introduction

Breast cancer is the leading cause of cancer-related mortality in women^1^. While estrogen receptor- (ER), progesterone receptor- (PR), and human epidermal growth factor receptor-2- (HER2) positive breast cancers have established therapeutic guidelines^2^, therapy of triple-negative breast cancer (TNBC) remains challenging without targeted treatment options^3–5^. Treatment normally involves chemotherapy combined with surgery and radiation, while emerging targeted therapies and immunotherapy are being investigated^5,6^. Anthracycline-based regimens are standard for TNBC management, preferred approaches include doxorubicin (DOX)-cyclophosphamide (CP), or epirubicin-CP, administered over four cycles spanning 8 to 12 weeks, followed by a taxane for an additional four cycles^7,8^. DOX, carboplatin (CPL), and CP combination is under investigation for its efficacy in neoadjuvant setting^8^.

Despite higher pathological complete response (pCR) rates, patients with TNBC experience significantly worse overall survival (OS) than those with non-TNBC breast tumors^9^. Disease recurrence remains a critical concern, with relapse rates typically peaking approximately three years post-TNBC diagnosis^10^. Intrinsically resistant cells or rare residual tumor cells can endure treatment and persist through a prolonged minimal residual disease (MRD) stage^11–13^. While resistant cells continue to proliferate despite cytotoxic therapy, drug-tolerant persister (DTP) cells undergo non-genetic adaptations, entering a dormant or slow-proliferating state^14^. DTP cells display diverse phenotypes, including metabolic changes, epithelial-to-mesenchymal transition (EMT), and activation of various detoxification pathways^15^. Over time, DTPs may transition into fully resistant populations, so early intervention is critical to disrupt this progression^16^.

Preclinical research on MRD primarily depends on in vitro and ex vivo systems, mouse models, and patient-derived xenografts^17,18^. Genetically engineered mouse models (GEMMs) can replicate human malignancies, including genetic profiles, interactions with the tumor microenvironment, and therapeutic responses^19^. BRCA1 mutation increases the likelihood of developing TNBC^20^. The BRCA1-deficient *K14cre;Brca1*^*F/F*^*;Trp53*^*F/F*^ (KB1P) mouse model has significantly advanced our understanding of therapy resistance mechanisms in BRCA1-mutated breast cancer^21^. Using Cre/loxP technology, this GEMM induces deletions of the *BRCA1* and *Trp53* genes within keratin 14-positive epithelial cells, leading to the development of spontaneous mammary tumors that mimic human BRCA1-mutated cancers^22^. Like TNBC patients, tumor-bearing KB1P mice initially respond to chemotherapy but eventually acquire resistance to multiple treatments^23–25^. Three-dimensional cancer-organoids derived from KB1P tumors^26^, enable genetic manipulation and clinically relevant mechanistic studies on therapy response and resistance^24,26,27^.

Accurate assessment of the residual tumor burden in experimental systems remains a major challenge when relying on traditional sampling methods. In contrast, non-invasive monitoring techniques in animal models offer several advantages: they are easy to implement, minimize stress to the animals, and enable real-time, rapid, and dynamic tracking of tumor kinetics^28^. Bioluminescence-based imaging can facilitate non-invasive preclinical imaging with single-cell resolution^28^. Directed evolution of firefly luciferase led to the development of the AkaLuc system, which generates in vivo emissions that are 100–1000 times brighter than those of conventional systems, allowing non-invasive visualization of single cancer cells trapped within the lungs of mice^29^. While bioluminescence imaging has certain limitations—for example, the signal can decrease when tumors are very large—AkaLuc remains highly efficient during early stages of tumor growth or relapse, when tumor burden is low^30^. To complement in vivo imaging, AkaLuc is often paired with a fluorescent reporter, such as mCherry, which allows parallel tracking of cancer cells by conventional methods, including flow cytometry and histochemistry. In this study, we apply a clinically used combination chemotherapy regimen for TNBC patients to track tumor cells through minimal residual disease (MRD) and relapse (REL) in mice bearing organoid-derived BRCA-deficient mammary tumors. By using an enhanced, non-invasive BLI system, we were able to monitor rare tumor cells over time. Our findings underscore the utility of this advanced BLI approach for tracking residual disease and reveal a critical role for the immune system in shaping tumor cell plasticity and adaptive responses to chemotherapy.

## Results

### A clinically relevant chemotherapy treatment protocol achieves sustained MRD and predictable relapse-free survival in organoid-derived mammary tumors

**Fig. 1:**
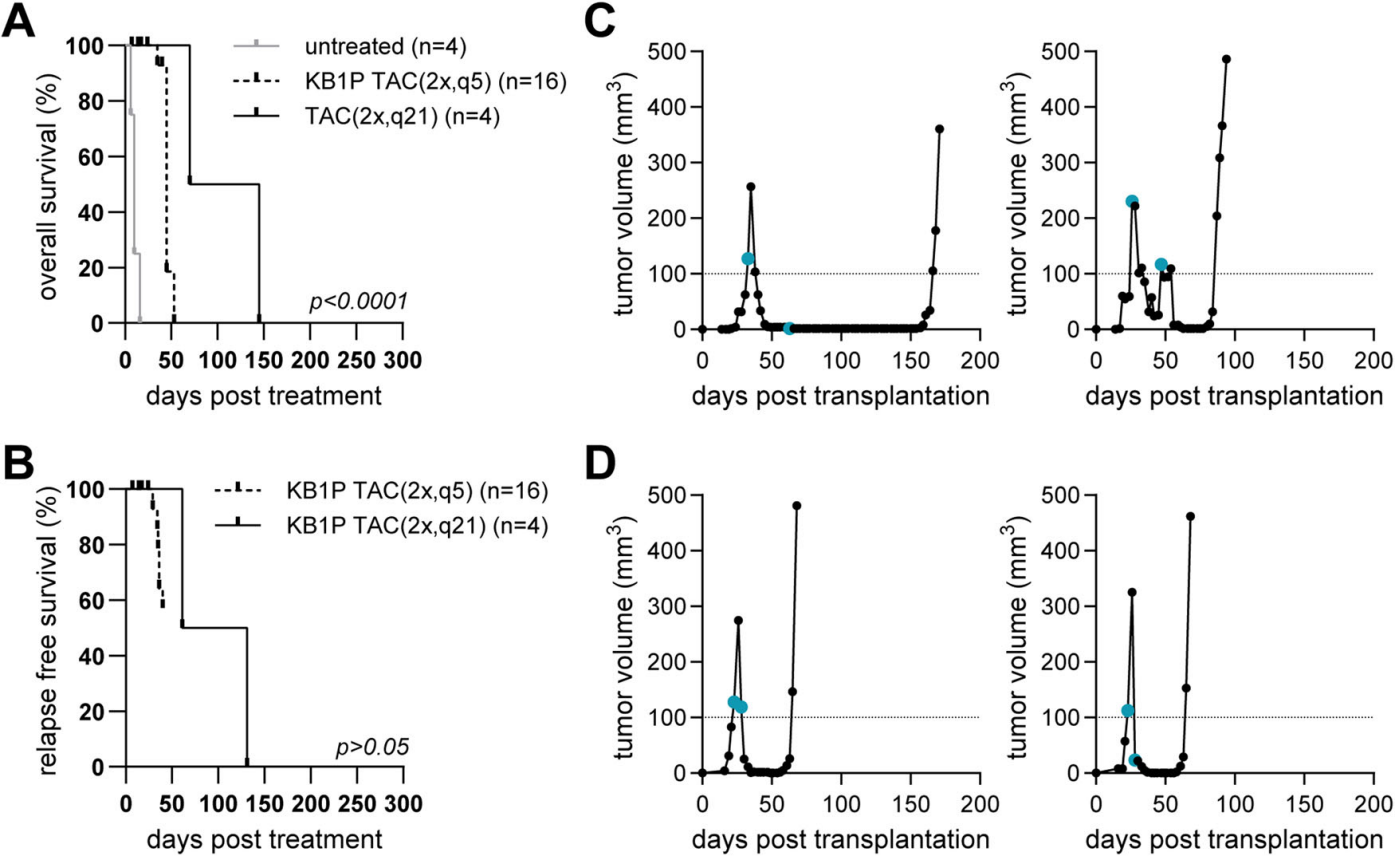
Establishment of a clinically relevant chemotherapy protocol inducing a stable MRD in the syngeneic KB1P organoid-derived tumor model. **A** Overall survival (OS) of untreated (gray) and TAC treated tumor-bearing FVB/N mice, second treatment separated by 21 (2x,q21; solid line) or 5 days (2x,q5; dashed line). **B** Relapse-free survival (RFS) of KB1P-transplanted FVB/N mice, treated with the two different TAC protocols. **C**, **D** Representative tumor growth and drug response curves of KB1P-transplanted FVB/N mice, treated with the **C** TAC(2x,q21) or **D** TAC(2x,q5) protocols. **C, D** turquoise circles on the tumor growth curves mark treatment days.

To model clinical therapy response in breast cancer, mouse mammary tumors, derived from KB1P organoids^26^, were treated with chemotherapy. Orthotopic transplantations of KB1P organoids into the 4th mammary fat pad of syngeneic FVB/N female mice showed reproducible growth kinetics (Supplementary Fig. S1A) that matched the original description of the model^26^. Organoid-derived tumors were treated with DOX according to previously established protocols for the KB1P model^24^. Though DOX therapy increased the median overall survival (OS) of KB1P tumor-bearing mice (Supplementary Fig. S1B), it did not significantly decrease tumor size (Supplementary Fig. S1C). Treatment with the liposomal formulation of DOX (pegylated liposomal DOX; Doxil Caelyx®) is effective even against DOX refractory KB1P tumors^24^. Accordingly, treatment with Doxil led to a significant increase of the median OS of tumor-bearing mice compared to DOX treated animals (Supplementary Fig. S1B). Albeit several mice treated with Doxil showed complete response, the onset and duration of the MRD phase varied (Supplementary Fig. S1D). Next, we adapted a clinically used combination treatment, consisting of DOX, DTX, and CP (TAC protocol)^31^. Two TAC treatments separated by 21 days induced a stable MRD phase in every tumor bearing mouse, with increased OS and a median relapse-free survival (RFS) of 96 days (Fig. 1A, B; TAC(2x,q21)). However, the onset of the relapse remained unpredictable, with some tumors relapsing before the administration of the second TAC cycle (Fig. 1C). Thus, the TAC protocol was modified by administering the second treatment cycle earlier, after 5 days (TAC(2x,q5)). While the higher initial cumulative drug dose of this treatment protocol resulted in decreased OS and RFS compared to the TAC(2x,q21) protocol (Fig. 1A, B), the short-term efficacy of the treatment increased without additional toxicity. Importantly, the TAC(2x,q5) protocol resulted in reproducible induction of a stable MRD phase lasting 30–40 days from treatment start in 70% of the animals (Fig. 1B, D).

### KB1P organoids engineered to express an mCherry-AkaLuc dual reporter confirm the increased sensitivity of the AkaLuc/AkaLumine system in vitro

**Fig. 2:**
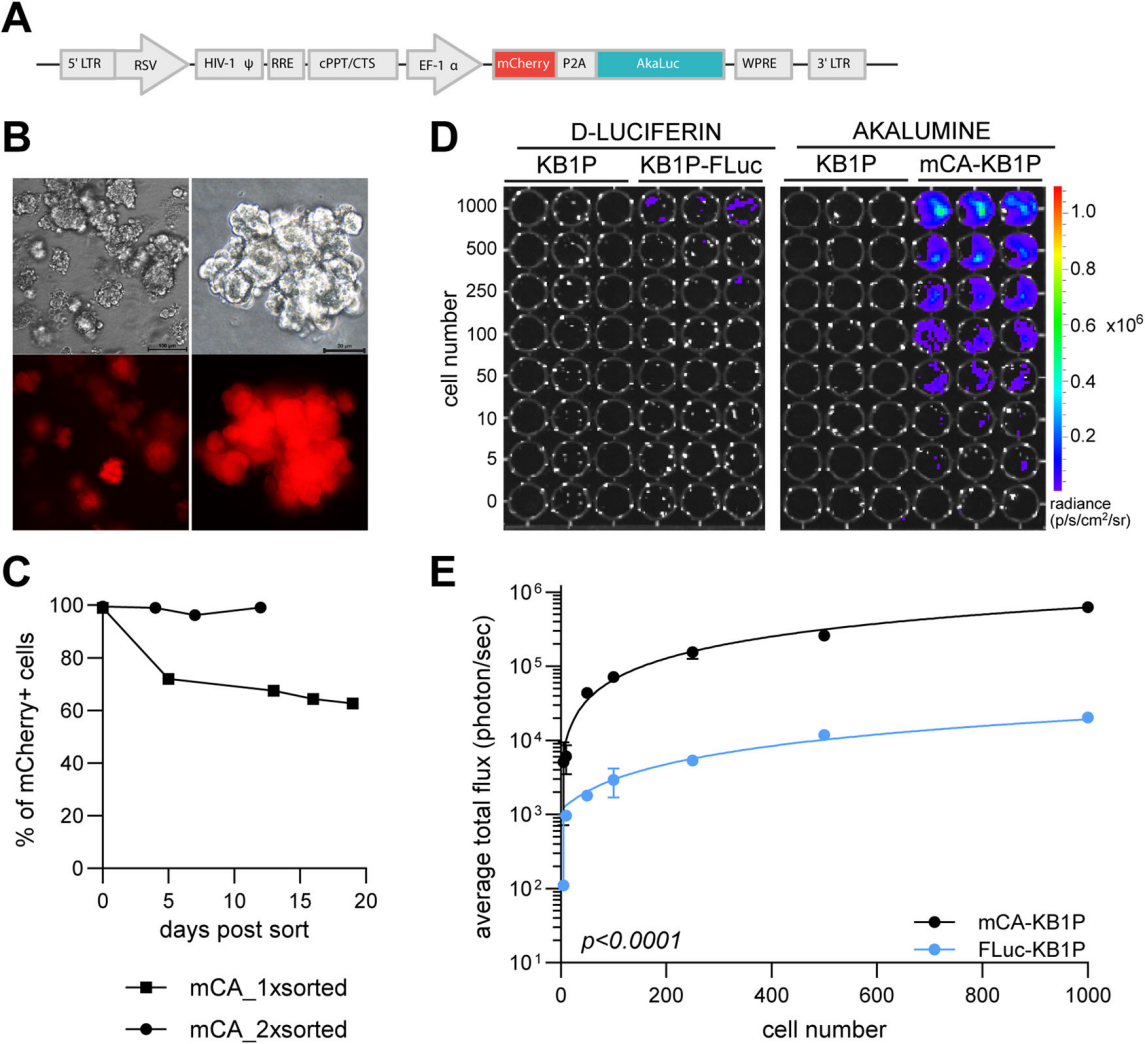
Generation and in vitro detection sensitivity of the dual mCherry-AkaLuc reporter system in KB1P organoids. **A** Schematic representation of the mCherry-AkaLuc lentiviral reporter construct. **B** Representative bright field and fluorescence microscopy images of KB1P organoids stably transduced with the mCherry-AkaLuc reporter (mCA-KB1P). Scale bar, 100 µm (left panel) and 20 µm (right panel). **C** Expression of the reporter over time following one or two rounds of FACS sorting of mCA-KB1P organoids. **D** Representative images of bioluminescence intensity (shown by the color scale) with increasing cell numbers (indicated by the numerical values on the left) for KB1P organoids expressing Fluc or AkaLuc. Wells containing parental organoids served as negative controls. Number of replicates for each condition: n = 3. **E** Quantification of in vitro bioluminescence expressed as total flux per cell [p/s/cell] plotted against the number of plated cells. Two-way ANOVA was used for statistical analysis.

To detect the few surviving cells at MRD, we aimed to establish high-sensitivity bioluminescence as an intravital imaging tool. To this end, a bicistronic lentiviral dual reporter was constructed expressing AkaLuc^29^ and mCherry (Fig. 2A). Fluorescence microscopy and fluorescence-activated cell sorting (FACS) analysis confirmed the stable expression of the reporter construct in transduced KB1P organoids (mCA-KB1P) after two rounds of enrichment by FACS (Fig. 2B, C). In vitro luciferase assays validated AkaLuc expression and confirmed a 100-fold increased sensitivity of the AkaLuc/AkaLumine system compared to Firefly luciferase (FLuc)/Luciferin^32^ (Fig. 2D). The lowest tested cell number of 5 cells could be detected with AkaLuc/AkaLumine compared to 500 cells for KB1P organoids engineered with FLuc. This enhanced sensitivity remained consistent with increasing cell numbers and appeared independent of the cell line used (Fig. 2E, Supplementary Fig. S2).

### An adapted TAC chemotherapy protocol for immunodeficient hosts enables the study of rare surviving MRD cell populations despite AkaLuc immunogenicity

**Fig. 3:**
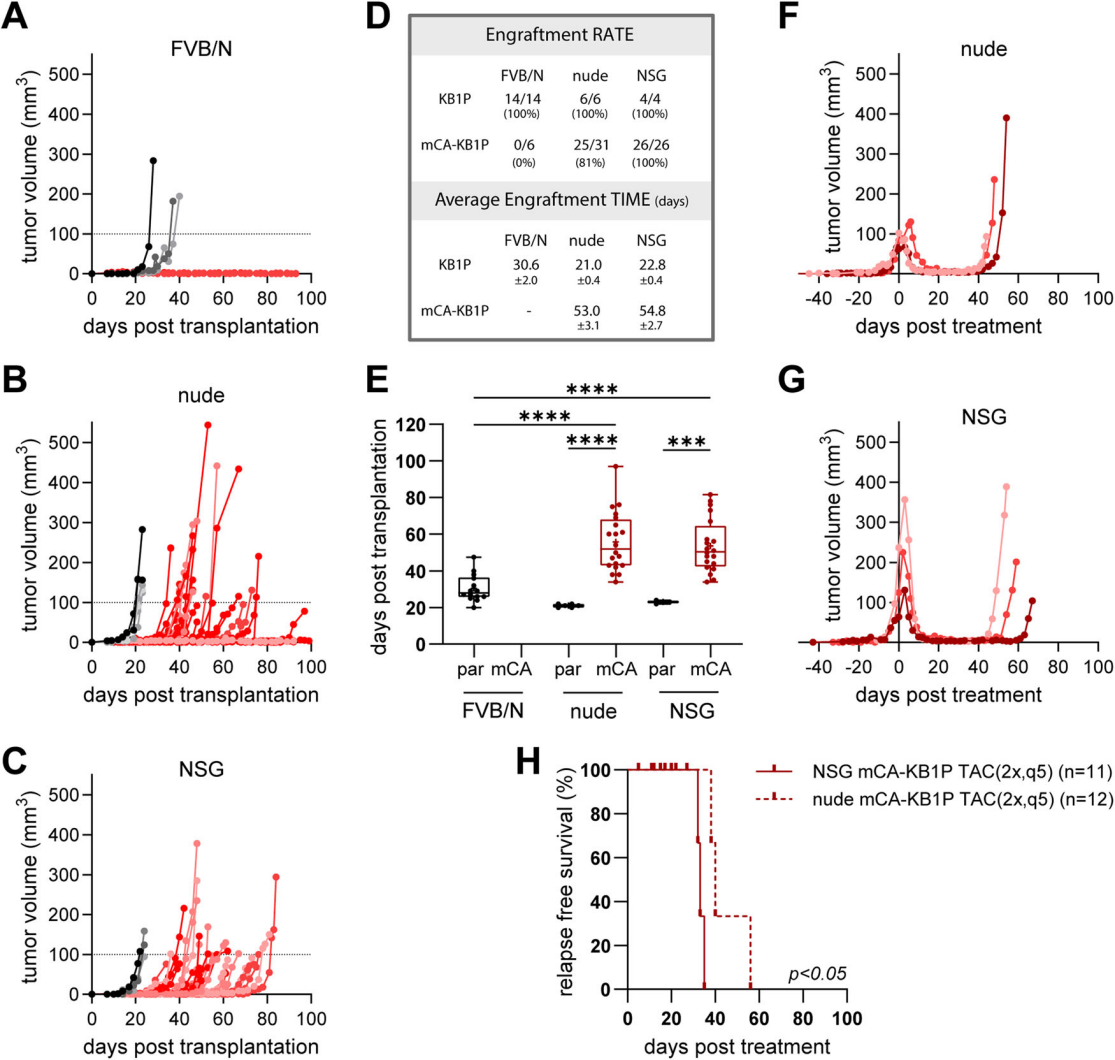
Immunodeficient mice support mCA-KB1P tumor engraftment and enable modeling of MRD following adapted TAC chemotherapy. **A–C** Tumor growth curves obtained in **A** FVB/N, **B** nude and **C** NSG mice transplanted with parental KB1P (gray shaded lines) or mCA-KB1P (red shaded lines) organoids. **D** Statistics of the engraftment rate (%) and time (days) of the transplants. **E** Median engraftment time (days needed after transplantation to reach treatment size). Statistical analysis was performed by One-way ANOVA. Significant differences are marked with asterisks **** *p* < 0.0001; *** *p* < 0.001. “par” and “mCA” mark tumor transplants with KB1P parental and mCA-KB1P organoids, respectively. (**F**, **G**) Tumor growth and drug response of **F** nude and **G** NSG mice transplanted with mCA-KB1P organoids and treated with the TAC(2x,q5) protocol **H** Relapse-free survival after therapy start of nude and NSG mice transplanted with mCA-KB1P organoids.

The validated AkaLuc reporter paved the way for monitoring the few tumor cells that survive chemotherapy during MRD in living animals using BLI. Unexpectedly, mCA-KB1P organoids failed to engraft in syngeneic FVB/N mice (Fig. 3A). To determine whether this was due to a loss of tumorigenic capacity or a potential immunogenic reaction of the host against the AkaLuc reporter, we transplanted parental KB1P and mCA-KB1P organoids into immunocompromised nude and immunodeficient NSG mice (Fig. 3B, C). mCA-KB1P organoids efficiently formed tumors in both immunodeficient strains (Fig. 3D). This strongly suggests an immunogenic characteristic of the AkaLuc protein, which has not been reported previously. The moderate engraftment rate in nude mice (81%) compared to NSG mice (100%), and the significantly delayed tumor outgrowth in both strains relative to the parental line in immunocompetent FVB/N hosts (Fig. 3D, E), further suggests a complex immune surveillance mechanism targeting AkaLuc, which includes, but is not restricted to, T cell–mediated immunity.

Next, we tested the established TAC(2x,q5) protocol in nude and NSG mice. We first optimized the drug dosing, with strain-specific maximum tolerated dose (MTD) and existing treatment regimens as guidelines^33–36^. Nude mice showed similar drug tolerability to syngeneic FVB/N hosts, allowing treatment with the same drug dosing. mCA-KB1P tumors in nude mice showed a complete response, characterized by a stable MRD phase, followed by relapse (Fig. 3G), similar to parental tumors in syngeneic FVB/N hosts (see Fig. 1D). NSG mice tolerated a substantially lower dosing of DOX and DTX. Nevertheless, the NSG-adapted TAC(2x,q5) protocol successfully recapitulated the treatment response of KB1P tumors observed in the other strains. All tumor-bearing NSG mice showed a stable MRD of reproducible length (20 ± 5 days) (Fig. 3F), albeit median RFS was significantly shorter than in nude mice (33 vs. 44 days) (Fig. 3H). This protocol enabled the monitoring of rare surviving tumor cells during the MRD phase in the mCA-KB1P organoid model in NSG mice using AkaBLI.

### Enhanced AkaLuc bioluminescence imaging of surviving tumor cells during MRD

**Fig. 4:**
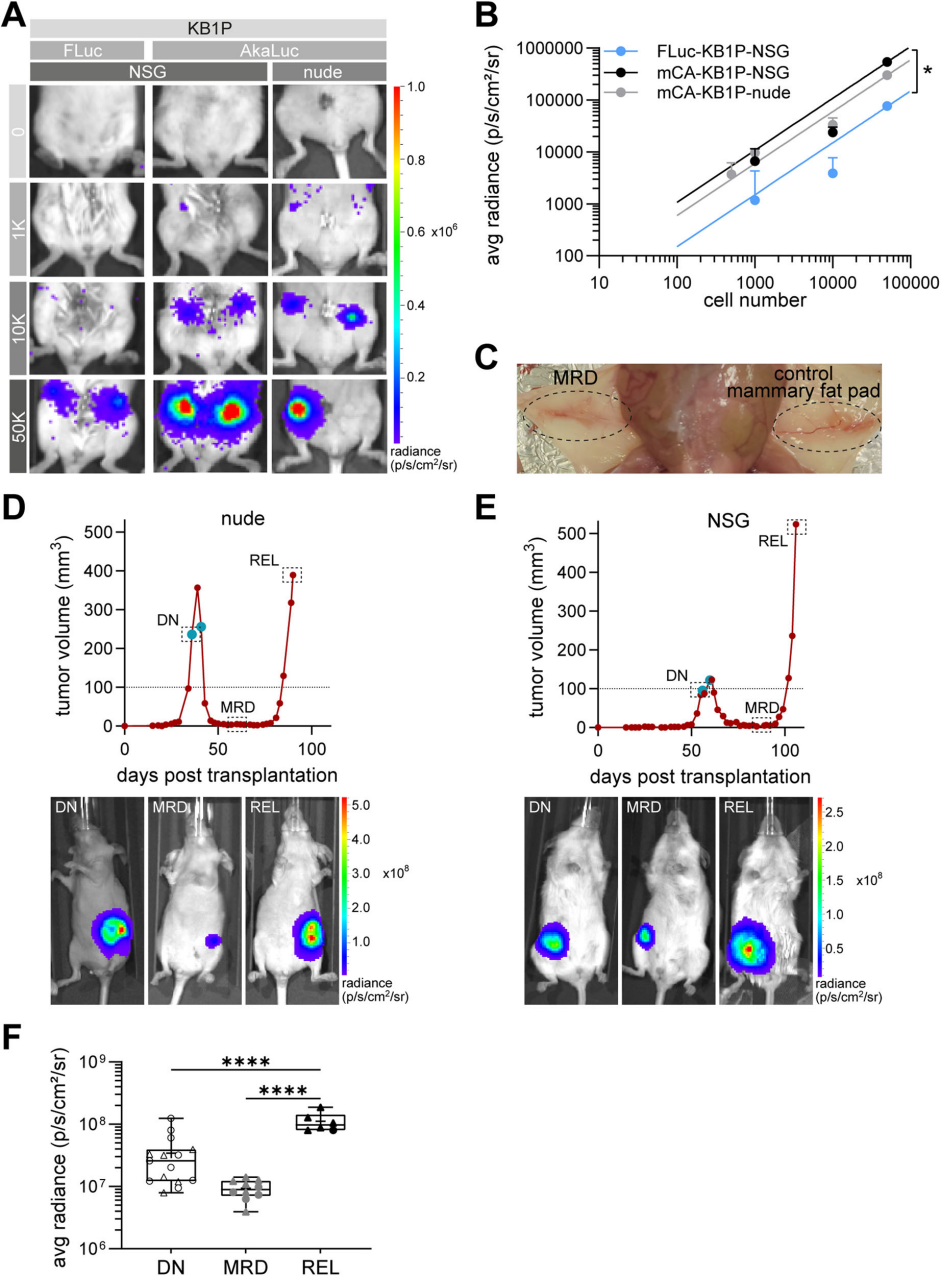
AkaLuc bioluminescence imaging enables detection of surviving tumor cells during minimal residual disease. **A** Representative bioluminescence images of mice transplanted with increasing numbers of KB1P organoids expressing Fluc or AkaLuc. **B** Quantification of bioluminescence intensity across different transplanted cell numbers in various mouse strains (mCA-KB1P- nude: 0 cells n = 4, 500 cells n = 4, 1 K cells n = 4, 10 K cells n = 4, 50 K cells n = 5; mCA-KB1P-NSG: 0 cells n = 6, 1 K cells n = 4, 10 K n = 4, 50 K n = 12; Fluc-KB1P-NSG: 0 cells n = 6, 1 K cells n = 2, 10 K cells n = 4, 50 K cells n = 4), represented as mean radiance +SEM relative to transplanted cell numbers. Two-way ANOVA was used for statistical analysis. Significant difference is marked with asterisk, (* p < 0.05). **C** Representative macroscopic image of the right 4th mammary fat pad of a nude mouse at the non-palpable MRD stage after mCA-KB1P organoid transplantation. The contralateral organ is shown as non-transplanted control. **D** and **E** Representative growth curves and drug response of mCA-KB1P organoid-derived tumors of a **D** nude and **E** NSG mouse treated with the TAC(2x,q5) treatment protocol. Treatment days are indicated by turquoise dots. Representative images of longitudinal BLI at drug naïve (DN), MRD, and relapse (REL) stages are shown under the corresponding tumor curves, framed data points mark the respective timepoints of BLI. **F** Quantification of BLI signal intensity as mean radiance of mCA-KB1P tumor-bearing nude and NSG mice at DN, MRD and REL stages (nude n = 1–9, NSG n = 5–6 per time point). One-way ANOVA was used for statistical analysis for combined data of nude and NSG mice. Data points for nude and NSG mice are shown as circles and triangles, respectively. Significant differences are marked with asterisks, (**** p < 0.0001).

Our in vivo mammary gland transplantation assay determined a detection limit of approximately 1000 cells for AkaBLI (Fig. 4A, B). This was tenfold lower than the 10,000 cell detection limit observed for KB1P organoids engineered with FLuc (Fig. 4A, B), although still moderate compared to the previously described single-cell sensitivity^29^. A hepatic background signal reported to limit the AkaLumine system^37–40^, was consistently observed across mouse strains (Supplementary Fig. S3). However, this did not affect our results, as the 4^th^ mammary fat pad does not overlap with the hepatic region.

AkaBLI enabled robust detection of 50,000 transplanted mCA-KB1P organoids (Supplementary Fig. S4A) and confirmed complete eradication of these cells in immunocompetent FVB/N hosts (Supplementary Fig. S4A–C). Interestingly, in approximately 20% of nude mice that did not develop tumors even after 110 days, persistent BLI signals were observed (Supplementary Fig. S4A), indicating long-term survival of transplanted tumor cells. The stable intensity of the initial BLI signal (Supplementary Fig. S4B, C), combined with IHC analysis showing low proliferation and apoptosis activity (Supplementary Fig. S4D), supports a state of tumor dormancy, likely maintained by residual immune surveillance.

To monitor BLI signal intensity changes over chemotherapy response, mice transplanted with mCA-KB1P organoids were imaged (i) as soon as tumors reached treatment size (DN stage), (ii) at stable MRD, and (iii) at tumor relapse (REL). Importantly, the MRD stage showed a prolonged period during which tumors were non-palpable (Fig. 4C), yet yielded detectable BLI signals in all mice. Figure 4D and E show a representative BLI image series aligned with the TAC(2x,q5) treatment response of mCA-KB1P tumors in nude and NSG mice, respectively.

BLI signal intensity and tumor cell numbers based on titration experiments (Fig. 4A), correlated with tumor volumes ranging from 0 and 150 mm³ (r = 0.4906, *p* < 0.01 and r = 0.5966, *p* < 0.001, respectively) (Supplementary Fig. S5A, B), most likely larger tumor volumes being influenced by necrotic and cystic cores. Therefore, data from tumors exceeding 150 mm³ were excluded from statistical analysis. (Supplementary Fig. S5C). The 0–150 mm^3^ tumor cohort showed distinct BLI intensities for the three different tumor stages (average radiance of 3 × 10^7^ in the DN state, approximately 1 × 10^7^ at MRD, and 1 × 10^8^ at REL (Fig. 4F)). Similar stage-dependent differences were observed in the corresponding tumor cell numbers (Supplementary Fig. S5D).

Average radiance levels showed no substantial strain-specific differences; however, significantly lower BLI intensities were observed in the DN compared to the REL stages (Fig. 4F), despite comparable tumor volumes (Supplementary Fig. S3C). While an up to 25-fold reduction of the tumor volume in the MRD phase was associated with only a 5-fold reduction in BLI signal intensity, the 25-fold increase in tumor volume in the REL phase corresponded to a significantly higher, up to 10-fold, increase in BLI signal intensity (Fig. 4F). These results suggest stage-dependent differences in the efficiency of bioluminescence signal generation. Indeed, DTP cells generated in the in vitro DTP repopulation assay^15^ using mCA-MCF7 cells and DOX treatment exhibited a significant, 4-fold lower bioluminescence signal intensity at DN stage compared to DTP cells (Supplementary Fig. S5D), supporting the notion that drug-induced phenotypic changes increase the efficiency of bioluminescent signal generation.

### HmC-KB1P organoids engraft in syngeneic immunocompetent FVB/N hosts with preserved drug response and stable MRD

**Fig. 5:**
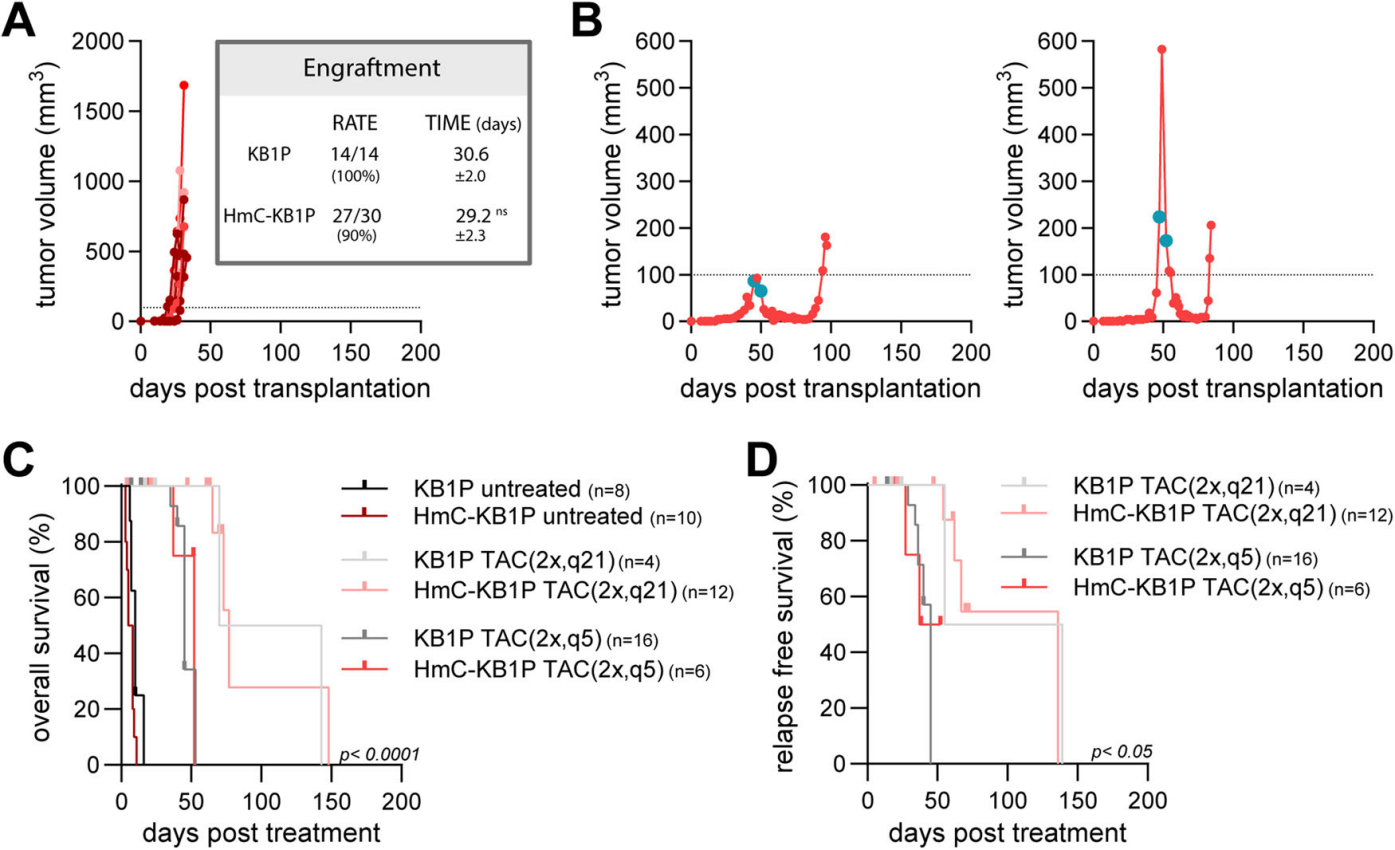
KB1P organoid-derived tumors engineered for HmC reporter expression engraft in syngeneic immunoproficient FVB/N hosts with maintained characteristics of drug response and stable MRD. **A**, **B** Representative growth curves of orthotopically transplanted HmC-KB1P organoid-derived tumors in female FVB/N mice **A** untreated or **B** treated with the TAC(2x,q5) chemotherapy protocol. Turquoise dots on the tumor growth curves mark the days of TAC treatment. The inset shows engraftment rates and median engraftment times; statistical comparison was performed using unpaired two-tailed students *t*-test. ns, non-significant. **C** OS of untreated (dark lines) and TAC-treated mice using 2x,q21 (light lines) or 2x,q5 (faded lines) protocols. **D** RFS of transplanted mice, treated with the two TAC protocols.

To enable the detection of surviving tumor cells after chemotherapy in immunocompetent hosts, H2B-mCherry (HmC) was introduced as a fluorescent reporter gene into KB1P organoids by lentiviral engineering (HmC-KB1P). HmC-KB1P organoids in syngeneic FVB/N hosts showed comparable engraftment rate, growth kinetics and drug response to the parental KB1P model (Fig. 5A, B). Consistently, tumors from HmC-KB1P organoids showed similar OS and RFS rates to parental KB1P organoids with both TAC(2x,q21) and (2x,q5) protocols (Fig. 5C, D). TAC(2x,q5) therapy response was also similar to mCA-KB1P organoids in nude and NSG mice (Fig. 3H and Supplementary Table S1).

### MRD is associated with reduced adaptive selection pressure in immunocompromised mice

**Fig. 6:**
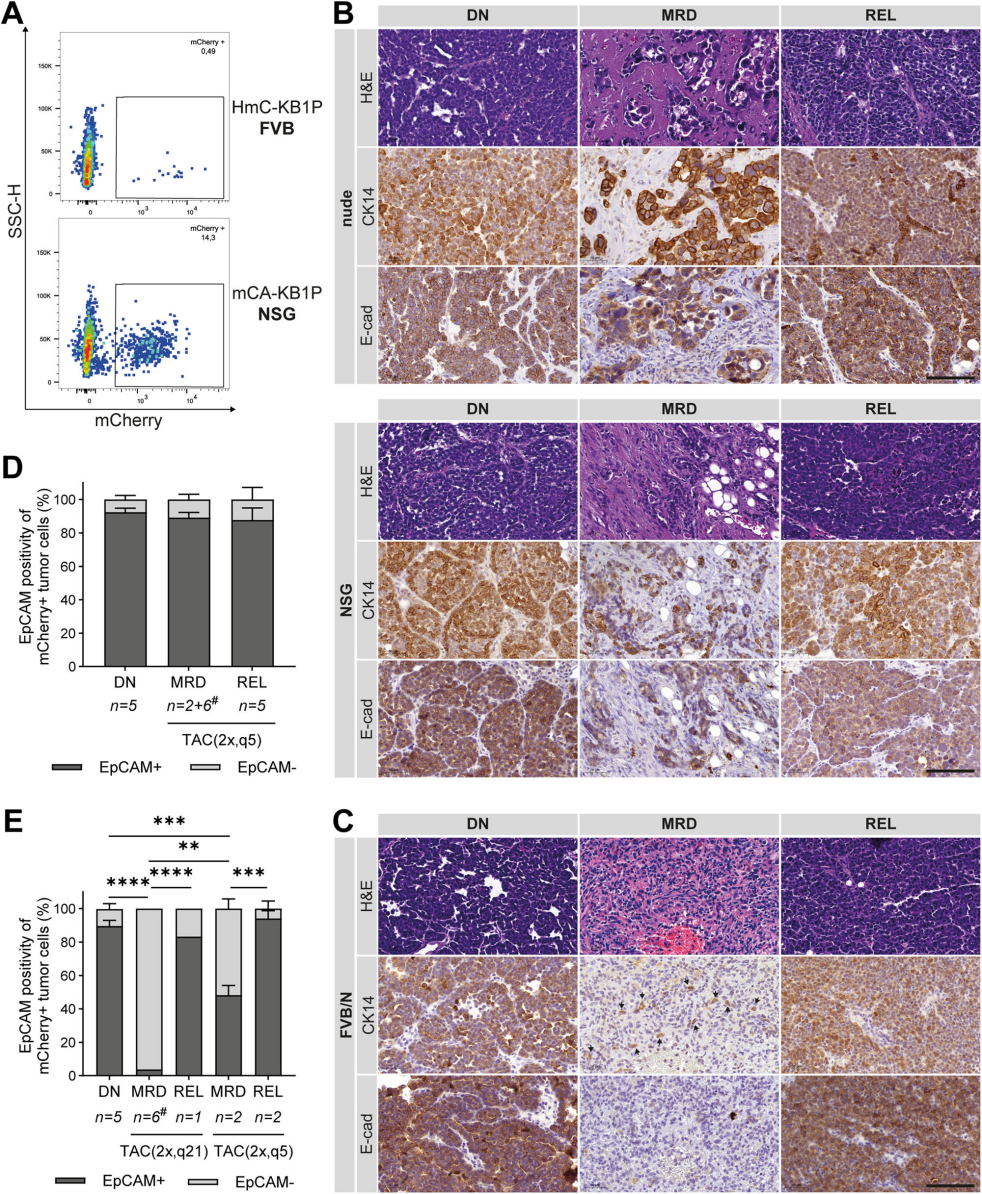
The MRD stage following combination chemotherapy is associated with reduced selection pressure in immunocompromised mice. **A** Quantification of tumor cells (mCherry + ) onFACS plots of MRD tumors isolated from FVB/N (top panel, TAC(2x,q21), n = 6 pooled) and NSG (bottom panel, TAC(2x,q5), n = 6 pooled) hosts. **B**, **C** Representative microscopic images of DN, MRD, and REL **B** mCA-KB1P tumor tissues isolated from nude and NSG or **C** HmC-KB1P tumor tissues from FVB/N hosts, stained with Hematoxylin-Eosin (H&E) and for Cytokeratin 14 (CK14) and E-cadherin (E-cad) expression. Arrows mark CK14-positive tumor cells. Scale bars, 100 µm. **D** and **E** Percentage ± SEM of EpCAM positivity of mCherry+ tumor cells in DN, MRD and REL of **D** mCA-KB1P tumors in immunodeficient and **E** HmC-KB1P tumors in FVB/N mice over the TAC treatment protocol. Number of mice used for the analysis is indicated under each bar. Nude and NSG hosts were combined for this analysis (DN: nude n = 1, NSG n = 4; MRD: nude n = 4, NSG n = 4; REL: nude n = 2, NSG n = 3). #: 6 samples were pooled for a single FACS analysis. Two-way ANOVA was used for statistical analysis to compare differences between groups. Asterisks mark significant differences, **** p < 0.0001, *** p < 0.001, ** p < 0.01.

To quantify surviving tumor cells at MRD, six HmC-KB1P and six mCA-KB1P tumors were harvested and pooled at the MRD stage from FVB/N and nude or NSG hosts, respectively and analyzed by flow cytometry using mCherry expression for cancer cell identification (Fig. 6A), along with the exclusion of immune and endothelial cells to account for differences in the tumor microenvironment (Supplementary Fig. S6A, B). The proportion of surviving tumor cells was markedly lower in immunocompetent compared to immunodeficient hosts during the MRD phase (0,47% vs. 14,5% of the CD45-,CD31- double-negative live cell population, respectively). This suggests that a very small number of cells survive treatment in immunocompetent mice, serving as a reservoir for tumor relapse. In contrast, significantly larger population of tumor cells survives chemotherapy and persists through the MRD phase in immunodeficient mice. Histological analysis confirmed these observations (Fig. 6B, C). While well detectable tumor cell islands were observed in the tumor stroma of hematoxylin-eosin (H&E) stained mammary fat pads in both nude and NSG hosts at the MRD stage (Fig. 6B), the residual tumors in FVB/N mice showed high accumulation of stroma with only a few surviving tumor cells scattered as solitary cells within the tumor stroma, as evidenced by cytokeratin 14 (CK14) immunostaining (Fig. 6C). Interestingly, during MRD, KB1P tumor cells retained expression of the epithelial marker E-cadherin in immunodeficient hosts (Fig. 6B), whereas at least half of the rare surviving tumor cells in FVB/N hosts showed a loss of E-cadherin expression (Fig. 6C). Epithelial plasticity, including epithelial-to-mesenchymal transition (EMT) contribute to therapy resistance and cancer cell survival after therapy^41^. To analyze this further, flow cytometry analysis was extended to include the epithelial marker EpCAM (Supplementary Fig. S6C, D). In immunodeficient mice, the majority of tumor cells (mCherry + ) was also positive for EpCAM throughout the DN, MRD, and REL phases (Fig. 6D). In contrast, while tumor cells in FVB/N mice exhibited similar EpCAM expression during the DN stage, the few surviving cells at MRD following TAC(2x,q21) treatment were negative for EpCAM. The TAC(2x,q5) protocol also resulted in significantly reduced EpCAM positivity at MRD, although this effect was less pronounced than with the TAC(2x,q21) protocol (Fig. 6E). At the REL stage, the majority of cancer cells reverted to an EpCAM+ epithelial phenotype following both protocols (Fig. 6E), suggesting that a substantial subset of the few surviving tumor cells in immunocompetent hosts had undergone a transient loss of epithelial characteristics. However, it should be noted that the small number of mice investigated in the REL phase limits the strength of the conclusions that can be drawn from this observation.

## Discussion

The KB1P mouse model is a well-established and clinically relevant system for studying BRCA1-deficient triple-negative breast cancer (TNBC)^22,42^, offering valuable insights into tumor biology, treatment response, and resistance mechanisms. Its use has significantly advanced our understanding of BRCA1-driven tumorigenesis and therapeutic vulnerability. However, like all preclinical models, it has certain limitations. Tumors in KB1P mice are relatively homogeneous and develop rapidly, which may not fully capture the genetic and phenotypic diversity or progression dynamics of human TNBC. Metastasis occurs inconsistently, and the model’s specificity for BRCA1-deficient TNBC limits its relevance to other subtypes, even within the broader TNBC category. The inherent sensitivity of these tumors to DNA-damaging agents, while beneficial for studying minimal residual disease (MRD), can restrict the generalizability of results to less therapy-sensitive tumor types.

Despite its limitations, the KB1P model has proven to be a valuable tool for studying treatment resistance in TNBC^21,23^. These tumors show initial sensitivity, but eventually acquire resistance to various drugs, providing an ideal preclinical model for the study of drug resistance evolution. In contrast to previous studies employing orthoptic transplantation of tumor pieces derived from KB1P tumors^22,25^, DOX treatment in our study did not result in a consistent reduction in the size of organoid-derived tumors. Since the organoids were established from treatment-naïve tumors, the observed resistance to DOX treatment may be attributed to genetic drift or phenotypic adaptation during in vitro cultivation. Notably, treatment response was improved by Doxil therapy, supporting its potential use in DOX refractory tumors^24^. Most prior studies utilizing the KB1P have focused on monotherapies^22,25,42^, even though standard clinical care for TNBC patients typically involves combination therapy, often based on anthracycline-containing regimens^7^. In this study, we adapted a clinically relevant TAC combination regimen^31^, which significantly prolonged RFS in mice bearing KB1P organoid-derived tumors. This therapeutic response closely mirrors clinical outcomes observed in TNBC patients^43^, highlighting the model’s translational relevance.

Whereas the administration of two TAC cycles within 5 days resulted in a complete response, tumors relapsed after 30–40 days, indicating that a small reservoir of cancer cells persisted at the primary tumor site following treatment. In this context, “complete response” refers to the absence of a palpable tumor, in line with clinical definitions that describe complete response as the disappearance of all detectable disease by physical examination or imaging, while recognizing that microscopic residual disease may still be present. This small reservoir of cancer cells—whether remaining at the primary tumor site, circulating in the bloodstream, or disseminated to distant organs—poses a significant challenge to achieving complete remission^44–46^, and is strongly associated with unfavorable clinical outcomes^47^.

To enable sensitive tracking of residual cancer cells, we established mCA-KB1P organoids. Unexpectedly, these organoids failed to engraft in FVB/N mice, likely due to an immunogenic response against AkaLuc. Supporting this hypothesis, AkaBLI imaging confirmed the elimination of mCA-KB1P organoids in FVB/N hosts. Interestingly, in a subset of nude mice that did not develop tumors, the engrafted mCA-KB1P organoids persisted in a dormant state for several months, suggesting that T cells play primary role in the elimination of AkaLuc-expressing KB1P cells in FVB/N mice. In contrast, all transplantations led to successful tumor formation in severely immunodeficient NSG mice, indicating that immune cell populations present in nude but absent in NSG mice —such as B cells, NK cells, and components of the complement system—may contribute to the immune response against the AkaLuc reporter protein by promoting tumor cell dormancy, independently of T cell activity. The use of nude and NSG mice necessitated the adaptation of the triple-combination TAC chemotherapy protocol, as it had not been previously established for these strains. The multifaceted immunodeficiency of NSG mice is known to reduce their tolerance to certain cytostatic drugs^48^. Accordingly, NSG mice in our study tolerated lower doses of DOX and DTX. Nevertheless, they exhibited a stable MRD phase of reproducible duration following TAC treatment, albeit with a shorter median RFS compared to nude mice. This system enabled the investigation of chemotherapy responses across the DN, MRD, and REL phases, and validated the efficacy of the established TAC(2x,q5) treatment protocol in achieving a complete response with a stable MRD phase in the KB1P tumor model across multiple mouse strains, irrespective of their immune status. It will be interesting to compare the drug-tolerant persisters that constitute the minimal residual disease (MRD) following this clinically relevant combination therapy regimen with those arising after cisplatin monotherapy, as established in previous studies using the KB1P mouse model^25–27^.

Bioluminescence imaging leverages the catalytic activity of luciferase enzymes acting on luciferin substrates to produce light, which is then captured during imaging^49^. However, BLI is limited by shallow imaging depth, challenges in accurate signal quantification, and reduced spatial resolution^49,50^. AkaBLI represents a significant advancement in both imaging sensitivity and depth^29^. Our results confirm the superior sensitivity of the AkaLuc system: in vitro, we were able to detect as few as 5 cells, demonstrating approximately 100-fold greater sensitivity compared to FLuc. In vivo, the detection threshold corresponded to approximately 1000 cells, representing a tenfold increase in sensitivity relative to FLuc. As expected, differences in BLI signal intensities were observed between the DN stage and the MRD and REL stages. BLI of mCA-KB1P cells enabled the detection of DTP cells during the MRD stage, even when tumors were no longer palpable. Although titration experiments confirmed a correlation between tumor cell numbers and BLI signal intensity, caution is warranted when estimating the number of persister cells during MRD, due to stage-dependent variations in BLI output. One possible explanation for these variations is the significant metabolic burden imposed by ATP-dependent luciferases during the bioluminescent reaction^51^. This metabolic demand may influence BLI output as a result of chemotherapy-induced metabolic adaptations in tumor cells, though further investigation is needed to clarify this relationship. Supporting this hypothesis, our in vitro experiments with AkaLuc-expressing DTP cells revealed higher-then-expected BLI signal intensity following DOX treatment, suggesting that drug-induced, tumor cell-intrinsic factors can influence AkaBLI signal intensity.

Extensive phenotypic changes in DTP cells were also evident from the histological analysis of tumor sections corresponding to DN, MRD and REL stages. In immunocompetent hosts, only a few tumor cells were detectable at the MRD stage, and a negligible fraction of these expressed epithelial markers such as EpCAM and E-cadherin. This suggests that surviving tumor cells are extremely rare and undergo a transient loss of epithelial characteristics during MRD. In contrast, in immunodeficient (NSG or nude) hosts, significantly more tumor cells were present at the MRD stage, and these cells retained epithelial marker expression comparable to those observed in the drug-naïve and relapsed stages. It should be noted that differences in the observed DTP phenotypes may, at least in part, be attributed to the use of different reporter systems in the immunocompetent (HmC-KB1P) versus immunodeficient (mCA-KB1P) models. Nevertheless, the findings suggest that the immune system contributes to shaping tumor cell plasticity and epithelial-to-mesenchymal transition (EMT), thereby influencing tumor cell survival following chemotherapy.

Increasing evidence supports the view that DTPs exploit EMT as part of their adaptive response to therapy. In non-small cell lung cancer (NSCLC), cells treated with the epidermal growth factor receptor (EGFR) inhibitor gefitinib exhibited reduced E-cadherin levels and increased vimentin expression in resistant cells. Similarly, an enriched EMT signature in DTPs was demonstrated for EGFR-mutant lung adenocarcinoma cells^52^ and patient-derived melanoma models^53^. Furthermore, DTPs often upregulate EMT-related markers, including ZEB1, Twist, and Slug, following therapy^54–56^, which is associated with their survival and adaptability^57^. Similar EMT activation has been observed in HER2-amplified breast cancer cells treated with lapatinib, basal-like breast cancer cells exposed to PI3K/mTOR inhibitors, and paclitaxel-resistant lung or prostate cancer cells tolerating EGFR-TKIs^58–60^. These findings underscore that EMT is intricately linked to the adaptive plasticity of DTPs.

The evolving understanding of TNBC biology has led to the identification of new therapeutic targets, paving the way for the development of targeted therapies^5^. Nonetheless, challenges remain, particularly in identifying predictive biomarkers of response and overcoming therapeutic resistance. This study introduces a mouse model system that can be used to explore these critical aspects, supporting the pre-clinical evaluation of novel targeted therapies. A major current limitation in DTP research is the lack of reliable biomarkers to detect, track, and characterize this rare cell population, or to predict their potential for reactivation and tumor relapse. To improve clinical applicability, future studies will need to enhance detection sensitivity and establish robust correlations between MRD, DTP prevalence, and clinical endpoints such as progression-free and overall survival^61^.

## Methods

### mCA plasmid generation

The bicistronic dual-reporter lentiviral construct was generated using the NEBuilder HIFI DNA Assembly Cloning Kit (#E5520S, New England Biolabs) with the lentiviral vector pRRL-EF1a-WPRE^30^ as backbone, linearized with the restriction enzymes BamHI (R3138S, New England Biolabs) and SalI (R3136S, New England Biolabs). Vector pU6-(BbsI)_CBh-Cas9-T2A-mcherry-P2A-Ad4E4orf6^62^ was a gift from Ralf Kuehn (Addgene plasmid # 64222; http://n2t.net/addgene:64222; RRID:Addgene_64222) and was used for polymerase chain reaction (PCR) amplification of the mCherry-T2A. Vector pcDNA3 Venus-AkaLuc^29^ was provided by the RIKEN BRC through the National BioResource Project of the MEXT, Japan (cat. RDB15781) and was used for PCR amplification of the AkaLuc fragment. Sequence fidelity was confirmed after plasmid assembly by Sanger sequencing.

### Cell and organoid culture

HEK293FT (RRID: CVCL_6911) cells were used for lentivirus production. Parental and engineered 4T1^63^ and MCF7 (ATCC HTB-22) cells were used for in vitro and in vivo titration assays. Cell lines were maintained in Dulbecco’s modified Eagle medium (DMEM; HEK293FT and 4T1) or RPMI (MCF7) supplemented with 10% fetal bovine serum (FBS), and 1% penicillin/streptomycin. During transfection, cells were grown without antibiotics. Parental and engineered KB1P breast cancer organoids were cultured as described^26^. Cells and organoids were maintained at 37 °C with 5% CO_2_.

### Lentiviral production in HEK293FT cells

The mCA constructs were used to produce lentivirus in HEK293FT cells using the standard CaCl_2_ transfection method. Briefly, 6 × 10^6^ HEK293FT cells were seeded into T75 culture flasks. After 24 h, the second-generation lentiviral packaging vectors psPAX.2 and PMD2.G and the plasmid of interest (mCA-plasmid, pLenti.PGK.H2B-chFP.W^64^, or pHIV-iRFP720-E2A-Luc^34^) were slowly mixed with sodium-phosphate. The generated DNA-calcium phosphate precipitate was directly pipetted onto the cells, which were cultivated in antibiotic-free medium for a few hours. 24 h post transfection, fresh medium was added, and the cells were incubated for an additional 48 h. Finally, the supernatant containing the viral particles was collected and filtered with a 0.45 µm low protein binding filter. For viral concentration, the filtered supernatant was ultra-centrifuged at 25,000 rpm for 2 h at 4 °C, and the pelleted virus was resuspended in 1/100 of the original volume. Viral titers were determined using the abm qPCR Lentivirus Titration Kit (abm, #LV900) according to the instructions. psPAX2 and PMD2.G were a gift from Didier Trono (Addgene plasmid # 12260; http://n2t.net/addgene:12260; RRID:Addgene_12260 and Addgene plasmid # 12259; http://n2t.net/addgene:12259; RRID:Addgene_12259, respectively). pLenti.PGK.H2B-chFP.W^33^ was a gift from Rusty Lansford (Addgene plasmid # 51007; http://n2t.net/addgene:51007; RRID:Addgene_51007). pHIV-iRFP720-E2A-Luc^65^ was a gift from Antonius Plagge (Addgene plasmid # 104587; http://n2t.net/addgene:104587; RRID:Addgene_104587).

### Lentiviral transduction of cell and organoid lines

Concentrated mCA viral particles at a multiplicity of infection (MOI) of 25 were added to 9 × 10^4^ MCF-7 cells in a final volume of 1 mL cultivation media in one well of a 6-well plate. After 48 h incubation at 37 °C with 5% CO_2_, cells were expanded and further cultivated according to standard procedures.

Lentiviral transduction of KB1P organoids was performed according to Duarte et al.^26^ with the following modifications: concentrated viral particles were mixed to 5 × 10^5^ (mCA) or 1.2 × 10^5^ (HmC, or FLuc) single cells of KB1P organoids at a MOI 25 in a final volume of 500 µL ENR in one well of an ultralow adherent 24-well plate (Greiner Bio-ONE CELLSTAR, #391-3375), followed by spinfection for 1 h at 600 g, room temperature and incubation for 6 h (mCA) and 48 h (HmC, or FLuc), respectively, at 37 °C with 5% CO2. Thereafter, KB1P cells were seeded in a density of 3 × 10^4^ cells/40 µL matrix in BME:ENR (1:1), and further cultivated according to the original protocol.

Transduction efficiency of mCA, HmC, or FLuc was evaluated by flow cytometry. To enrich for transduced cells, Fluorescence Activated Cell Sorting (FACS) was applied, followed by flow cytometric validation of enrichment. Finally, reporter expression was evaluated by microscopy.

### Mice and mammary fat pad transplantation

Mice were kept in the animal facility of the Medical University of Vienna in accordance with institutional policies and federal guidelines. Animal experiments were approved by the Animal Experimental Ethics Committee of the Medical University of Vienna and the Austrian Federal Ministry of Science and Research (animal license number: BMBWF 2020-0.121.294 and BMBWF 2024-0.518.920). Organoids were transplanted as described earlier^26^. Briefly, organoids were used at a size corresponding to an average of 150–200 cells per organoid structure. Organoid suspensions containing a total of 5 × 10^4^ cells in 30 µl of organoid media/BME mixture (1:1) were injected orthotopically into the fourth right mammary fat pad of 7-week-old wild-type FVB/N (KB1P, HmC-KB1P, mCA-KB1P), NMRI nude (mCA-KB1P), and NSG (mCA-KB1P) mice (Charles River Laboratories, Germany) under anesthesia (100 mg/kg ketamine and 5 mg/kg xylazine) after a small surgical incision. The tumor size was monitored at least 3 times per week by caliper measurements. Tumor volumes were calculated using the formula V = 0.5 × length × width^2^. Animals were sacrificed at the experimental endpoint (200–300 mm^3^ for drug-naïve, at MRD, or 200–2000 mm^3^ for relapse samples). For engraftment experiments mice were sacrificed before the tumor volume reached 2000 mm^3^.

### Chemotherapeutics and treatment of tumor-bearing mice

DOX (Adriblastin; 2 mg/ml), pegylated liposomal DOX (Doxil) (Caelyx®; 2 mg/ml), Docetaxel (DTX; Accord Healthcare; 20 mg/ml), and CP (Endoxan; 20 mg/ml) were obtained directly from the manufacturers in clinically used formulations and were further diluted in saline before use to a final injection volume of 200 µL.

The triple-combination therapy TAC was performed in FVB/N mice as described earlier for other tumor models^31^ with modifications. Briefly, the drugs were used in following doses: docetaxel 25 mg/kg, DOX 5 mg/kg and CP 120 mg/kg. First, CP was administered by intraperitoneal (i.p.) injection, which was followed by an intravenous (i.v.) injection of a freshly prepared mix of DTX and DOX. Therapy was started either at a tumor volume of 200 ± 50 mm^3^, including two treatment cycles separated by 21-days (TAC(2x,q21)); or at 100 ± 25 mm^3^, with two cycles separated by 5 days (TAC(2x,q5)). Nude mice were treated with the same dose. In the case of NSG mice, doses were reduced as follows: DTX 7.5 mg/kg, DOX 1.5 mg/kg and CP 120 mg/kg. For both strains therapy was administered at a tumor volume of 100 ± 25 mm^3^, in two treatment cycles, separated by a 5-day-interval.

### Bioluminescence imaging

For in vitro evaluation of the AkaBLI sensitivity in the mCA-KB1P organoids, an in vitro titration assay was combined with BLI. Parental KB1P, mCA-KB1P and FLuc-KB1P organoids and parental MCF7, parental 4T1, mCA-MCF7 and FLuc-4T1 cells were trypsinized to single cells and serial dilutions of 5, 10, 50, 100, 250, 500 and 1000 cells were seeded in PBS/0.5% BSA in triplicates into a black 96-well imaging plate with a glass bottom (Eppendorf, #0030741030). AkaLumine (#013-23693, FUJIFILM Wako Chemicals Europe GmbH) substrate solution [500 µM] or D-Luciferin (#7902, BioVision) substrate solution [99 mM], was added to the cells in a final concentration of 250 µM. Bioluminescence measurements were performed after 15 min of substrate incubation as described previously^29^. The following settings were used for the IVIS® Spectrum System (Perkin Elmer): open for total bioluminescence, exposure time: 40 s (MCF-7, KB1P, mCA-MCF-7, mCA-KB1P), 60 secs (FLuc-KB1P), and 300 secs (4T1, FLuc-4T1), binning: 4 (medium), FOV: C, f/stop: 1, temp at measurement: 20 °C–25 °C.

To evaluate drug-induced intensity changes of AkaBLI, in vitro 2D repopulation assay was performed in parental and mCA-MCF7 cells as described^15^ and cells were harvested by trypsinization for BLI measurements at day 1 (drug naïve), and 22 and 29 (DTP). AkaBLI was measured in a plate reader (Tecan) after seeding 10,000 cells in PBS/0.5% BSA in triplicates into a black 96-well imaging plate with glass bottom (Corning) and immediate addition of AkaLumine in a final concentration of 250 µM with following settings: central wavelength of 648 nm with 105 nm bandwidth, integration time of 3000 ms and kinetic mode with four measurement cycles in two minutes intervals. Evaluation and quantification of the signal were carried out using the IVIS software’s built-in “Well Plate Quantification” tool. Background correction was appropriately taken into account by designating specific wells as “background wells,” as supported by the software’s functionality. Technical triplicates were averaged and a minimum of three independent biological replicates were measured.

In vivo titration analyses were performed to test the in vivo sensitivity of the bioluminescence of the FLuc- and mCA-KB1P organoids. Engineered KB1P organoids were trypsinized to single cells and serial dilutions of 1 × 10^2^, 5 × 10^2^, 1 × 10^3^, 5 × 10^3^, 1 × 10^4^, 5 × 10^4^, 1 × 10^5^, 2.5 × 10^5^, 5 × 10^5^ organoid cells were prepared in PBS on ice. A volume of 30 µL of these organoid-cell solutions was injected into the fourth left and right mammary fat pad of anaesthetized female nude, or NSG mice. The duration of the surgical procedure was standardized across all experimental groups, lasting 16 min for each batch of five mice. Directly after, 50 mg/kg AkaLumine-Hydrochloride (“TokeOni”, #HY-112641A, THP and #808350, Sigma Aldrich) substrate (30 mM in ddH2O) or 150 mg/kg D-Luciferin (#7902, BioVision) substrate [99 mM] solution was injected intraperitonially and 17 min after substrate administration bioluminescence measurements were performed at the IVIS® Spectrum System (Perkin Elmer) with the following settings: open for total bioluminescence, exposure time: 1 sec, binning: 4 (medium), FOV: D, f/stop: 1, temp at measurement: 20 °C–25 °C.

For in vivo detection of transplanted, engrafted, treated, and relapsed mCA-KB1P tumors, BLI was performed the day of transplantation, the day of drug treatment (75–150 mm^3^ tumor size), after chemotherapeutic treatment at MRD ( < 5 mm^3^ tumor size), and when the tumors relapsed (100–600 mm^3^ tumor size). BLI analysis was performed in Living Image (Perkin Elmer) software.

For BLI postprocessing and calculation of normalized cell numbers, images were loaded and adjusted to an ‘aggregate color scale’ for a uniform signal intensity interval, followed by marking the thoracic region per mouse for automatic background correction. ROIs for hepatic and mammary fat pad signals were marked manually and BLI intensities of each ROI calculated as ‘avg radiance [p/s/cm^2^/sr]’ after baseline correction by the hepatic and fat pad region of no substrate injected and 0-cell transplantation mice of the given strain, respectively. Normalized cell numbers at the different tumor stages were calculated by dividing these intensities by the slope (k) of the linear fitting curve of the corresponding in vivo cell titration.

### Tumor dissociation and flow cytometry

Tumor-bearing mice were sacrificed before the initiation of the treatment (Drug-Naïve, DN), at the MRD stage, or at relapse (REL). While DN and REL tumors could be dissected as well-defined tissue masses, in MRD samples a comparable, 1.5 cm long central piece of the mammary fat pad (transplantation site and area of tumor growth before treatment) around the inguinal lymph node was cut out, the adjacent normal tissue providing carrier cells for the preparation. Suspensions containing a high rate of viable cells were prepared as described^66^, with modifications. Briefly, excised tissue was cut into small pieces using scalpel blades, tissue fragments were shaken in Digestion Media (RPMI supplemented with 250 µg/ml DNase I and 1 mg/ml Collagenase IV) for 40 min at 37 °C. Digests were filtered through 70 µm strainers, washed with DNase I (100 µg/ml) containing washing buffer (PBS with 2% FCS), and centrifuged for 5 min (4 °C, 300 g). Following lysis of red blood cells (RBC lysis buffer, # B420301, Biozym) the pellet was washed twice in 10 ml Wash Buffer, centrifuged for 5 min (4 °C, 300 g). For flow cytometry, the resulting pellet was pre-blocked with anti-CD16/32 antibody (#156603, BioLegend) and stained with the following fluorophore conjugated primary antibodies for 30 min at 4 °C: CD31 (PE, #561073, BioLegend), CD45 (BV421, #103134, BioLegend) and epithelial cell adhesion molecule (EpCAM) (Fluorescein isothiocyanate [FITC], #118207, BioLegend), followed by Zombie Aqua viability dye (#77143, BioLegend) staining according to the manufacturer’s recommendation to exclude dead cells. Samples were analyzed by a Fortessa X20 (BD Bioscience) cytometer or by a FACSMelody cell sorter (BD Biosciences); data were analyzed using the FlowJo v10 software (BD Life Sciences).

### Immunohistochemistry (IHC)

Immunohistochemistry was performed according to Malik et al.^67^. In brief, following treatment with 3% H_2_O_2_ (Sigma Aldrich), 4 µm thick sections of mouse tumor tissues were repeatedly washed with 0.1% Tween 20 in PBS (PBS-T) and incubated in 5% horse blocking solution for 60 min in a wet chamber. Primary antibodies anti-E-cadherin (1:1000, #3195, Cell Signaling Technologies), anti-Cytokeratin 14 (1:500, #905303, BioLegend), anti-mCherry (1:250, #600-401-379S, ThermoFisher), anti-Ki67 (1:3000, #ab15580, Abcam) and anti-activated Caspase 3 (1:300, #9661S, Cell Signaling Technologies) were diluted in blocking buffer (2% BSA, 5% horse serum in PBS-T) and incubated overnight at 4 °C. Slides were washed and incubated with horseradish peroxidase (HRP)-conjugated secondary antibodies (1:2, #8114, Cell Signaling Technologies) for 60 min at room temperature, followed with 3,3´-diaminobenzidine (DAB substrate (DAKO)) for detection according to the manufacturer’s instruction.

### Statistical analysis

Statistical analysis was done with GraphPad Prism 10.04. No formal statistical method was used to estimate sample size. To compare one parameter between two groups, unpaired or paired two-tailed Student’s *t*-test were used, across multiple groups one-way or two-way analysis of variance (ANOVA) with Tukey multiple comparison test was applied to determine statistical significance. Overall and relapse-free survival (OS and RFS) were calculated using log-rank statistics. *P*-values less than 0.05 were considered statistically significant (*P < 0.05, **P < 0.01, ***P < 0.001). Error bars are represented as standard error of mean (SEM). The strength of association between two variables was calculated by Pearson correlation analysis, linear regression curves calculated and presented with 99% confidence intervals.

## Supplementary information

Steinbauer et al_Suppl File_revised

## Data Availability

No large datasets were generated or analyzed during the current study.
