# Supplementary material for: Enhanced bioluminescence imaging of tumor cells surviving chemotherapy in a murine model of triple-negative breast cancer
Source: NPJ Breast Cancer. 2025 Jul 30;11:80. doi: 10.1038/s41523-025-00795-y (PMC12311029; doi:10.1038/s41523-025-00795-y)
Supplement: Supplementary file 1 — Steinbauer et al_Suppl File_revised [file 41523_2025_795_MOESM1_ESM.pdf]

Enhanced Bioluminescence Imaging for Intravital Monitoring of Rare Tumor Cells Surviving Chemotherapy in a Murine Model of Triple-negative Breast Cancer

Silvia Steinbauer[1,#], Jamie D. Cowles[1,#], Mohammad Ali Sabbaghi[1], Marle Poppelaars[1], Azaz Hussain[1], Marina Wagesreither[1], Daniela Laimer-Gruber[2], Jozsef Tovari[3], Gergely Szakacs[1,*], Agnes Csiszar[1,*]

[1]Center for Cancer Research, Medical University of Vienna, Vienna, Austria

[2]Department of Biomedical Imaging and Image-Guided Therapy, Division of Structural and Molecular Preclinical Imaging, Medical University of Vienna, Vienna, Austria

[3]Department of Experimental Pharmacology and the National Tumor Biology Laboratory, National Institute of Oncology, Budapest, Hungary

[#]equally shared contribution

*corresponding authors:

gergely.szakacs@meduniwien.ac.at,

agnes.csiszar@meduniwien.ac.at

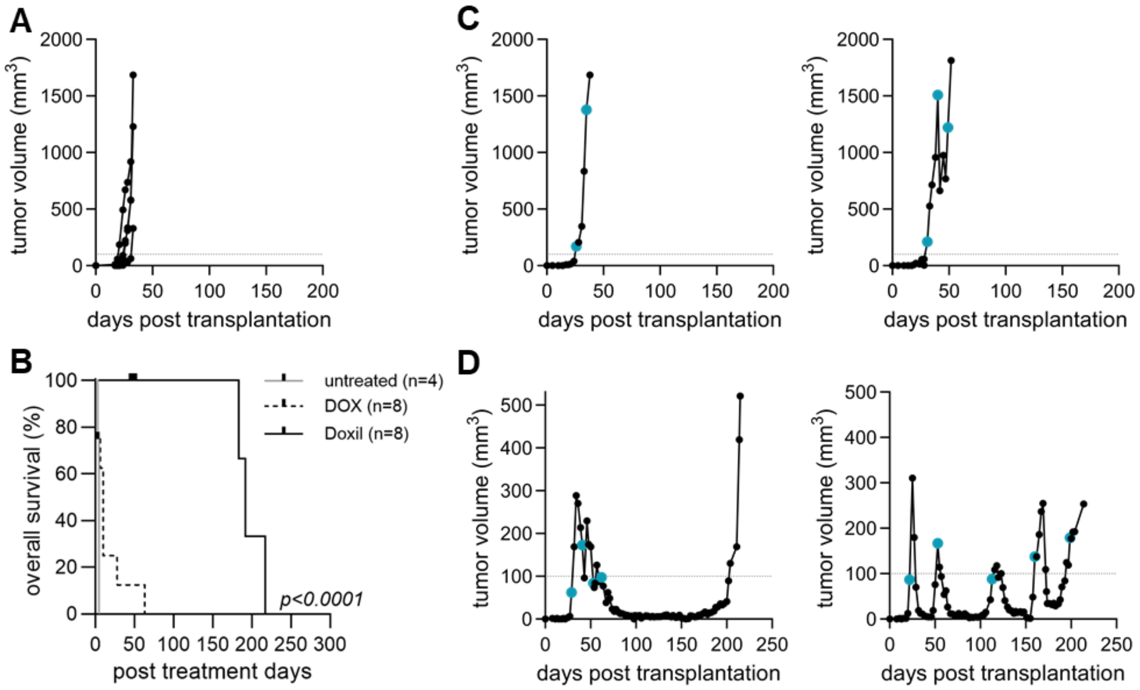

Supplementary Figure S1 (to Figure 1). DOX and Doxil treatment response of the syngeneic KB1P organoid-derived tumor model. (A) Representative tumor growth curves of untreated FVB/N mice after orthotopic transplantation with KB1P tumor organoids. **(B)** Overall survival (OS) of untreated (grey), DOX (dashed line) and Doxil (solid line) treated tumor-bearing FVB/N mice. **(C,D)** Representative tumor growth curves of **(C)** DOX and **(D)** Doxil-treated tumor-bearing FVB/N mice. **C,D** turquoise circles on the tumor growth curves mark treatment days with the respective drugs.

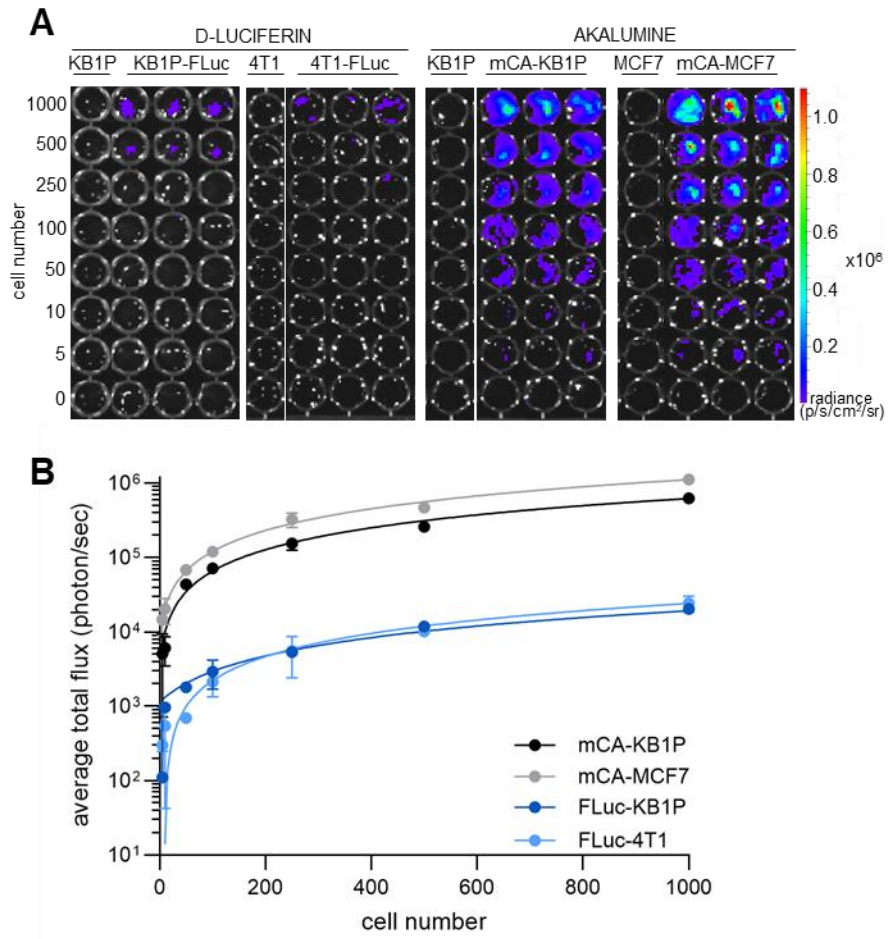

Supplementary Figure S2 (to Figure 2). Increased *in vitro* detection sensitivity of the dual mCherry-AkaLuc reporter system compared to the Fluc system is similar in other cell lines as well.

(A) Representative image of bioluminescence intensity (depicted by the color scale) with increasing cell numbers (indicated by the numerical values on the left) for KB1P organoids and 4T1 cells with Fluc, and KB1P organoids and MCF7 cells with AkaLuc expression. Wells containing parental organoids/cell lines served as negative controls. **(B)** Quantification of *in vitro* bioluminescence as total flux/cell [p/s/cell] as a function of plated cell number. To facilitate comparison, data from Figure 2D and 2E are re-included here as well.

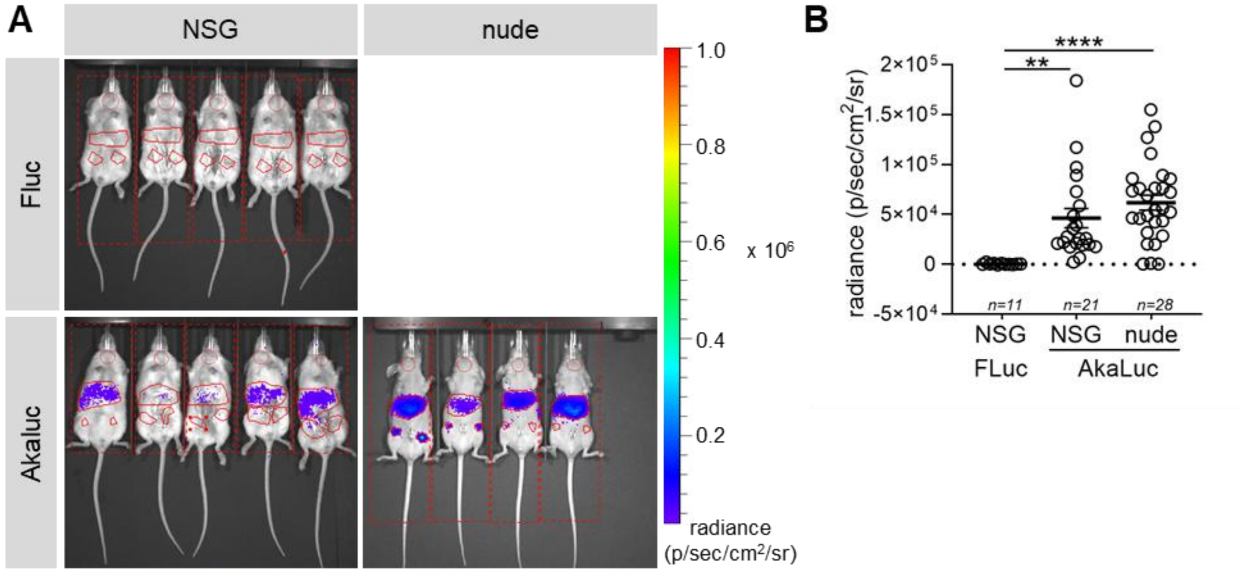

Supplementary Figure S3 (to Figure 4). The hepatic background of the Akaluc-AkaLumine BLI system limits the detection of rare tumor cells in overlapping anatomical regions.

(A) Representative whole-body bioluminescence images of NSG and nude mice from diverse transplantation experiments administered with D-Luciferin or AkaLumine substrates. Red frames indicate areas for signal quantification in the thoracic, hepatic and mammary gland regions.

(B) D-Luciferin and AkaLumine-derived background bioluminescence of the hepatic area quantified as mean radiance ± SEM in NSG and nude mice.

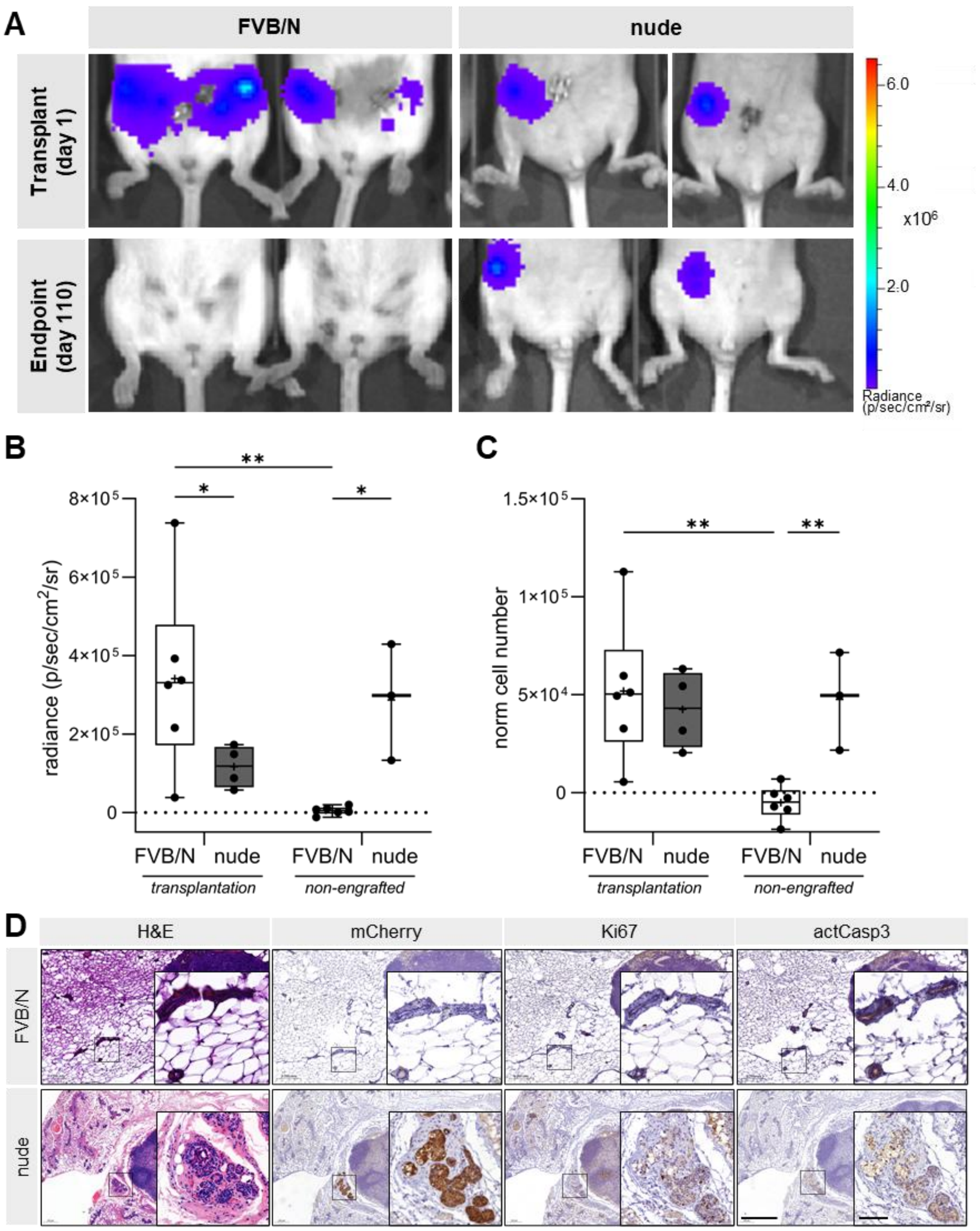

Supplementary Figure S4 (to Figure 4). Long-term BLI monitoring of mCA-KB1P tumor cells highlights a dormant survival of tumor organoid transplants in nude mice. (A) BLI images of representative FVB/N and nude mice transplanted with 50,000 mCA-KB1P tumor cells, captured at transplantation (top row) and after 110 days (bottom row). **(B,C)** Quantification on median radiance and corresponding calculated cell number of transplanted and non-engrafted tumor cells based on the dose-curve in Fig.4B. Two-way ANOVA was used for statistical analyses (FVB/N transplantation n=6, FVB/N non-engrafted n=6, nude transplantation n=4, nude non-engrafted n=3). Significant differences are indicated by asterisks, (** p<0.01, * p <0.05). **(D)** Representative microscopic images of histological sections of BLI-positive mammary fat pad regions of nude mice and comparable regions of FVB/N mice without tumor outgrowth 110 days after transplantation of mCA-KB1P tumor cells, stained with Hematoxylin-Eosin (H&E) and for mCherry, Ki67 and activated Caspase3 (actCasp3) expression. Scale bar, 500 µm, for inlet 100 µm.

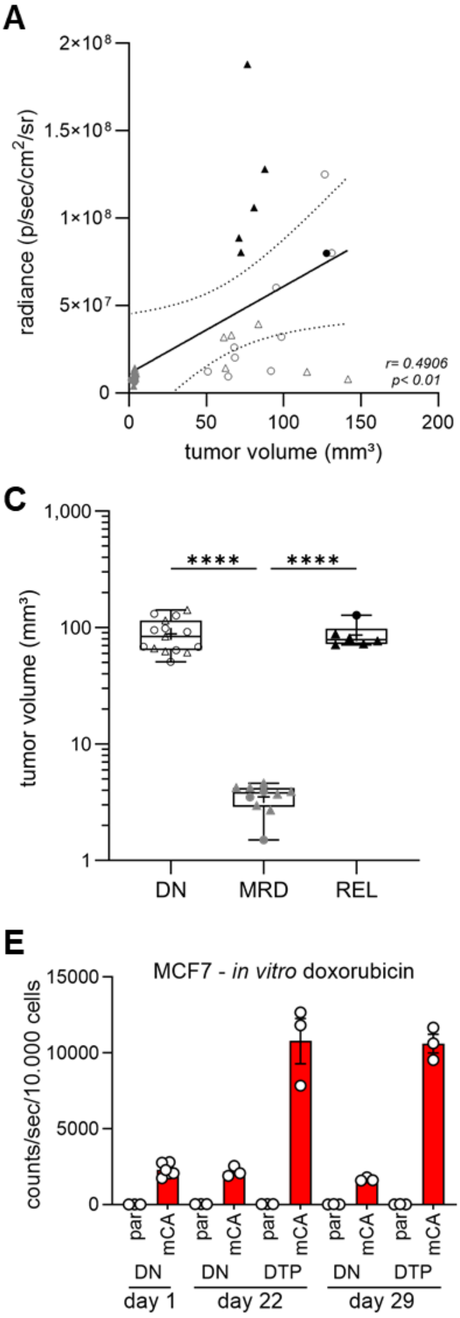

Supplementary Figure S5 (to Figure 4). (A,B) Pearson correlation analysis of tumor volume and corresponding **(A)** radiance or **(B)** tumor cell numbers calculated from BLI dose-titration. **(C,D)** Median **(C)** tumor volumes **(D)** and estimated tumor cell numbers calculated from the dose-titration experiments in (A) at DN, MRD and REL stages. **(A-D)** Statistics show combined values for nude and NSG mice. Circles and triangles mark nude and NSG data points, respectively. Empty symbols denote DN, grey symbols mark MRD, and black symbols show REL samples. **(E)** *In vitro* Akaluc-AkaLumine BLI of parental (par) and mCherry-Akaluc overexpressing (mCA) MCF7 cells at different time points of a 2D repopulation assay corresponding to drug naïve (DN) and drug-tolerant persister (DTP) cellular stages quantified as mean counts/sec ± SEM normalized to cell number. **(C,D)** One-way ANOVA with Turkey's multiple comparisons test was used for statistical analyses. Significant differences are indicated by asterisks, (**** p<0.0001).

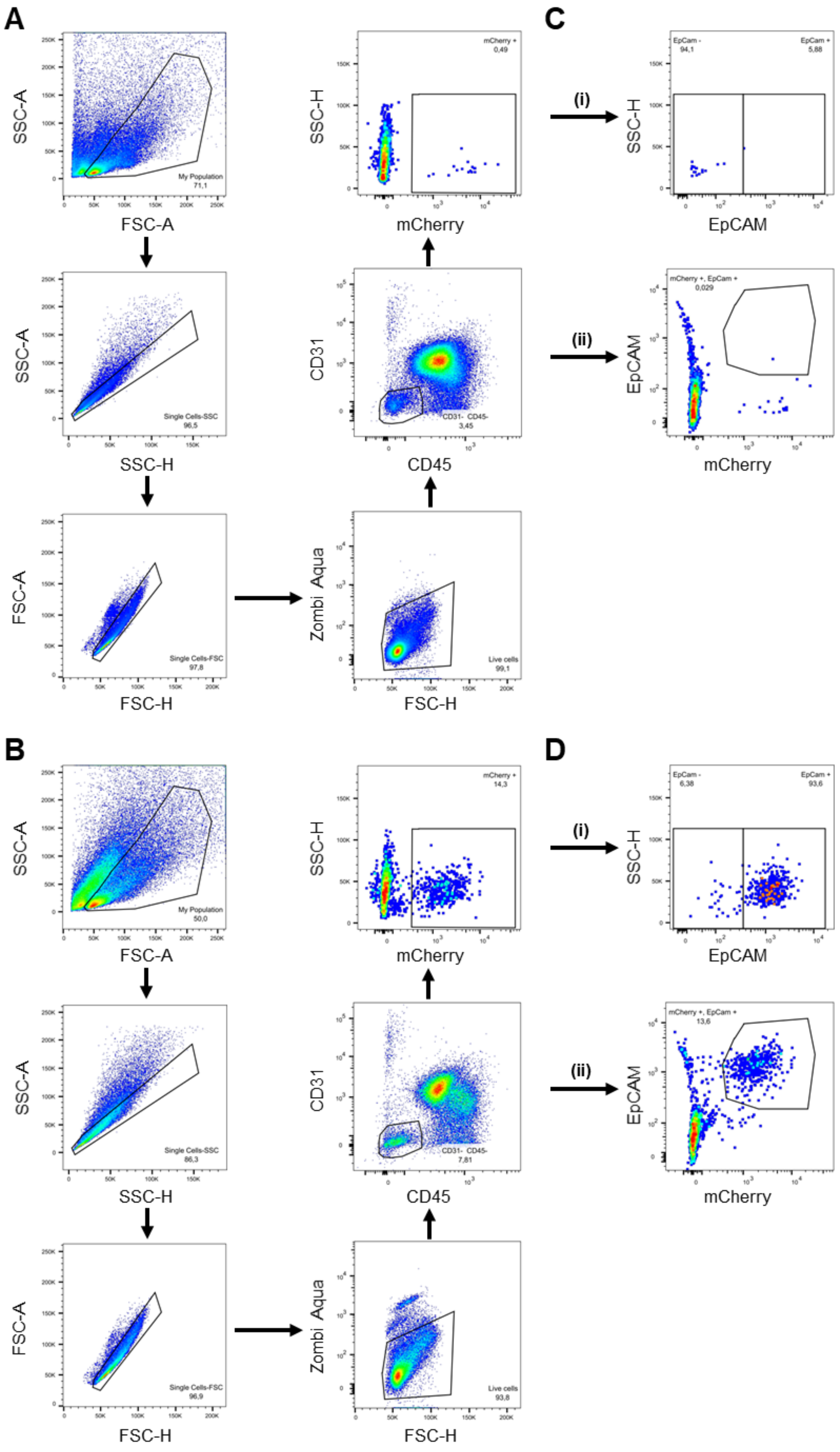
A
B
C
D
(i)
(ii)
SSC-A
FSC-A
SSC-H
FSC-H
Zombi Aqua
CD31
CD45
mCherry
EpCAM
My Population 71,1
Single Cells-SSC 96,5
Single Cells-FSC 97,8
Live cells 99,1
CD31- CD45- 3,45
mCherry + 0,49
EpCam - 94,1
EpCam + 5,88
mCherry +, EpCam + 0,029
My Population 50,0
Single Cells-SSC 86,3
Single Cells-FSC 96,9
Live cells 93,8
CD31- CD45- 7,81
mCherry + 14,3
EpCam - 6,38
EpCam + 93,6
mCherry +, EpCam + 13,6

Supplementary Figure S6 (to Figure 6). Gating strategy for the detection of mCA-KB1P and HmC-KB1P tumor cells and their EpCAM positivity in tumor single cell suspensions. (A,B) Tumor cell detection in **(A)** HmC-KB1P (FVB/N host) and **(B)** mCA-KB1P (NSG host) tumors: events after cell debris exclusion gated for single cell population followed by dead cell exclusion (Zombi Aqua+) and gating the CD45-,CD31- double-negative live cell population for mCherry expression. **(C,D)** EpCAM positivity of **(C)** HmC-KB1P (FVB/N host) and **(D)** mCA-KB1P (NSG host) tumor cells: (i) mCherry positive tumor cells gated for EpCAM expression and percentage of EpCAM- and EpCAM+ tumor cell sub-populations determined; (ii) CD45-,CD31- double-negative live cells gated for EpCAM and mCherry expression to quality control EpCAM immunostaining on mammary epithelial cells of the co-isolated adjacent normal tissue. **(A-D)** show representative MRD stage tumors, same settings were used for DN and REL stages.

Supplementary Table 1. Median OS and RFS (days) after different chemotherapy protocols in different mouse strains bearing organoid-derived KB1P mammary tumors

host	organoid	treatment								
		untreated	DOX		Doxil		TAC(2x,q21)		TAC(2x,q5)	
		median OS	median OS	median RFS	median OS	median RFS	median OS	median RFS	median OS	median RFS
FVB/N	KB1P	5	10	n.a.	192	n.a.	107.5	96	45	45
	HmC-KB1P	6.5	-	-	-	-	77	136	52	44.5
	mCA-KB1P	no engraftment								
NMRI nude	KB1P	-	-	-	-	-	-	-	-	-
	HmC-KB1P	-	-	-	-	-	-	-	-	-
	mCA-KB1P	10	-	-	-	-	-	-	59	40
NSG	KB1P	-	-	-	-	-	-	-	-	-
	HmC-KB1P	-	-	-	-	-	-	-	-	-
	mCA-KB1P	7	-	-	-	-	-	-	48	33

"n.a.": not adequate

"-": experiment not performed
